# Intrathecal Nalbuphine Versus Other Opioids as Adjuvant to 0.5% Hyperbaric Bupivacaine in Caesarean Section: A Systematic Review and Meta-Analysis

**DOI:** 10.3390/jcm15114108

**Published:** 2026-05-26

**Authors:** Anna Theodorou-Kanakari, Styliani Karathanasi, Panagis M. Lykoudis, Martina Rekatsina, Eleftheria Lelekaki, Maria Gavriilaki, Pinelopi Kouki

**Affiliations:** 1 Department of Anaesthesiology, General Hospital of Nikaia-Piraeus “Agios Panteleimon”, 18454 Athens, Greece; stellaaa_14@hotmail.com (S.K.); penkouki@gmail.com (P.K.); 2 Division of Surgery & Interventional Science, University College London (UCL), London WC1E 6BT, UK; p.lykoudis@ucl.ac.uk; 3 1st Anaesthesiology Department, Aretaieion University Hospital, National and Kapodistrian University of Athens, 11528 Athens, Greece; mrekatsina@gmail.com; 4 Intensive Care Unit, Naval Hospital of Athens, 11521 Athina, Greece; 5 1st Department of Neurology, AHEPA University Hospital, School of Medicine, Aristotle University of Thessaloniki, 54124 Thessaloniki, Greece

**Keywords:** caesarean section, duration of effective analgesia, hyperbaric bupivacaine, intrathecal nalbuphine, opioid adjuvants, spinal anaesthesia, systematic review, meta-analysis

## Abstract

**Background/Objectives**: Subarachnoid anaesthesia is widely preferred for caesarean delivery due to its rapid onset, reliability, and safety. The addition of adjuvants to intrathecal hyperbaric bupivacaine has been shown to enhance the quality of anaesthesia and prolong postoperative analgesia. Opioids are among the most frequently used intrathecal adjuvants; however, their administration is associated with adverse effects. Nalbuphine, a mixed opioid agonist–antagonist, may be a promising agent for obstetric use due to its favourable pharmacodynamic properties. This study aimed to systematically review and, where feasible, meta-analyse the evidence comparing nalbuphine with other intrathecal opioid adjuvants to hyperbaric bupivacaine 0.5% for caesarean section, with a primary focus on efficacy and safety. **Methods**: A systematic review and meta-analysis of controlled trials was conducted. Web of Science Core Collection, MEDLINE (PubMed), Scopus, the Library of Congress, and LISTA (EBSCO) were systematically searched to identify eligible studies. Pooled mean difference (MD) and risk ratio (RR) were calculated using random-effects model. **Results**: Ten studies encompassing 1095 parturients were included. Compared with fentanyl, intrathecal nalbuphine was associated with a modest prolongation of effective analgesia. However, this finding was accompanied by substantial heterogeneity and very low certainty of evidence. In contrast, morphine provided a longer duration of analgesia than nalbuphine. Overall, nalbuphine and fentanyl demonstrated comparable block characteristics, with only minimal and clinically insignificant differences in onset and duration. Importantly, nalbuphine was associated with fewer adverse effects, particularly shivering and PONV. **Conclusions**: Subarachnoid nalbuphine may serve as a potential alternative adjuvant to 0.5% hyperbaric bupivacaine in caesarean section anaesthesia. It may provide an analgesic efficacy comparable to fentanyl and, although less potent than morphine, it may demonstrate a more favourable safety profile.

## 1. Introduction

Caesarean section is included among the most frequently performed surgical procedures worldwide, with an increasing frequency over the last few decades [1,2]. Subarachnoid anaesthesia is one of the preferred techniques, as it provides high-quality sensory and motor block while avoiding foetal depressant effects, associated with general anaesthesia [3]. Hyperbaric bupivacaine is one of the most popular and potent local anaesthetics for intrathecal use [4]. It exerts its effects by blocking voltage-gated sodium channels on neuronal membranes, thereby increasing the threshold for action-potential initiation [5]. Nevertheless, there are doubts about the optimal dosage, especially when used as a single agent. In order to achieve adequate anaesthesia, high doses must be delivered, which can cause serious side effects, due to extended sympathetic blockade [6]. On the other hand, if lower doses are used, there is an increased risk for failure and conversion to general anaesthesia [7]. Therefore, addition of adjuvants has become common practice, since optimal perioperative conditions can be achieved, regarding quality and duration of blockade, along with prolongation of analgesia [8].

A wide range of drugs can be used as adjuncts to intrathecal hyperbaric bupivacaine for caesarean delivery, including opiates, ketamine, neostigmine, alpha-2-agonists, midazolam, and magnesium sulphate [9]. The most commonly used adjuvants are opioids, such as morphine and fentanyl. Specifically, morphine is widely considered the gold standard intrathecal adjuvant opioid for caesarean section [10]. Intrathecal opioids modulate pain transmission, both pre- and post-synaptically in the dorsal horns [11]. Activation of μ- and δ-opioid receptors leads to opening of G-protein-coupled potassium channels, while κ-receptor activation results in calcium channel closure [11]. Both mechanisms reduce intracellular calcium levels, inhibit release of excitatory neurotransmitters, and consequently induce potent antinociception [11]. Stimulation of μ-receptors by opioids is responsible for a wide range of dose-dependent adverse effects, such as nausea, vomiting, pruritus, urinary retention, sedation, and, most notably, delayed respiratory depression [12].

Nalbuphine is a semi-synthetic, lipophilic phenanthrene opioid, chemically related to oxymorphone and naloxone and acts primarily as a κ-receptor agonist with partial μ-receptor antagonistic effects [13,14]. Analgesia is mediated via κ-receptor activation, while μ-antagonism is expected to reduce the aforementioned opioids’ serious adverse effects [15]. Furthermore, it exhibits minimal addictive potential. Nalbuphine’s promising pharmacodynamic profile renders it an attractive option for obstetric use.

Opioid-related side effects can contribute to delayed ambulation and breastfeeding among parturients, prolongation of hospital stay and consequently maternal discomfort [16,17]. Anaesthesiologists often deal with the dilemma of choosing the optimal agent that will enhance anaesthesia blockade and prolong postoperative analgesia without exposing parturients to evitable risks.

The aim of this study was to systematically review evidence on the comparison of nalbuphine to other opioids as intrathecal adjuncts to hyperbaric bupivacaine, for caesarean section, mainly focusing on efficacy and safety. Existing reviews on intrathecal nalbuphine have not specifically addressed the obstetric population. Accordingly, the present study focuses on this specific clinical context. Primary endpoints included duration of motor and sensory block along with duration of analgesia, while secondary endpoints mostly involved incidence of adverse effects.

## 2. Materials and Methods

We have registered our systematic review on the PROSPERO database under the code number CRD42025632443 on 21 January 2025. The systematic review and meta-analysis were undertaken in accordance with the Preferred Reporting Items for Systematic Reviews and Meta-Analyses (Supplementary PRISMA 2020 Checklist) guidelines [18].

### 2.1. Search Strategy and Information Sources

Two researchers independently conducted a systematic search of the electronic literature to identify studies related to the administration of intrathecal nalbuphine as an adjuvant to 0.5% hyperbaric bupivacaine in caesarean section. The following databases were queried: Web of Science Core Collection, MEDLINE (PubMed), Scopus, Library of Congress, and LISTA (EBSCO). An initial search was performed on 22 January 2025, and the search strategy was structured using two blocks of keywords, with all possible combinations between them applied. Block A comprised the term “nalbuphine,” while Block B included the terms “spinal” OR “intrathecal” OR “subarachnoid” OR “regional.” Both controlled vocabulary terms (MeSH, LCSH, LISTA subject headings) and free-text keywords with truncation were used. The full Boolean strings for each database are presented in Appendix A to ensure transparency and reproducibility. The reference lists of the identified studies were also screened for additional eligible articles. A final search was conducted in the same databases on 23 January 2026 to identify more recent studies that had not been initially retrieved.

### 2.2. Eligibility Criteria

The present study was conducted in compliance with the PICO framework (P = Patient/Population, I = Intervention, C = Comparison, O = Outcome) as follows: “In obstetric patients undergoing caesarean section (P), does intrathecal nalbuphine (I) as an adjuvant to 0.5% hyperbaric bupivacaine, compared with other opioids (C), provide effective postoperative analgesia with fewer side effects(O)?”. Hence, the present review focuses on identifying studies that compared intrathecal administration of nalbuphine combined with 0.5% hyperbaric bupivacaine during caesarean section to other commonly administered opioids [19]. Studies were limited to those using 0.5% hyperbaric bupivacaine to maintain uniformity in the anaesthetic regimen and minimise variability in block characteristics and postoperative analgesic outcomes. Researchers applied predefined eligibility criteria to titles and abstracts in order to identify pertinent studies for inclusion. The selection criteria encompassed prospective randomised and non-randomised controlled trials, as well as retrospective studies, written in English, with accessible full texts. Included studies involved only adult parturients undergoing caesarean section under spinal anaesthesia where intrathecal nalbuphine (combined with 0.5% heavy bupivacaine) was compared specifically with other intrathecal opioid adjuvants. Articles available only as abstracts, case reports and non-comparative studies were excluded. Additionally, studies involving intrathecal nalbuphine combined with local anaesthetics other than 0.5% heavy bupivacaine or studies comparing nalbuphine with other non-opioid adjuvants were excluded. Studies administering nalbuphine via alternative routes, or those focusing on other types of operations were also eliminated. For the remaining articles, researchers independently reassessed inclusion criteria through full-text evaluation to determine their suitability. In cases of disagreement, a third author was consulted. For studies with multiple treatment arms (e.g., those including midazolam or placebo), only the data comparing nalbuphine with other opioids were utilised for the primary analysis to ensure conceptual homogeneity. Data regarding placebo or non-opioid comparators were recorded and presented for transparency and as supplementary clinical context, but did not influence the qualitative synthesis between opioid agents.

### 2.3. Data Collection Process and Outcome Measures

Data from the included articles were extracted by two researchers independently, using a structured Excel spreadsheet. Τhe results were cross-checked afterwards. In cases of disagreement, a third researcher resolved discrepancies. Extracted data included the first author’s name, year of publication, number of included patients, demographic characteristics (mean age in years, mean weight in kilograms, mean height in centimetres, ASA grade), as well as the dosage of hyperbaric bupivacaine and type of opioid which was co-administered. Primary outcomes included: (i) duration of effective analgesia (mean in minutes), defined as the time interval from intrathecal injection to first requirement of rescue analgesia; (ii) duration of motor block (mean in minutes), measured as time interval between the intrathecal administration and complete motor recovery (Bromage 0); and (iii) duration of sensory block (mean in minutes), identified as the interval commencing from intrathecal injection to regression of sensory level by 2-segments from the highest dermatomal block level. Secondary outcomes included: (i) onset of motor block (mean in minutes) measured as time elapsed from intrathecal injection until the point when the parturient could no longer move her feet (Bromage score 3); (ii) onset of sensory block (mean in minutes), determined by timing from subarachnoid injection until loss of pain sensation at T5 dermatome level; (iii) duration of complete analgesia (mean in minutes) specified as time interval from subarachnoid injection to the first sensation of pain (VAS > 0); and (iv) mean Apgar score at 1 min and mean Apgar score at 5 min. As additional secondary outcomes, the incidences of the following adverse effects were recorded: hypotension (percentage per arm), bradycardia (percentage per arm), postoperative nausea and/or vomiting (PONV) (percentage per arm), sedation (mean sedation score, Ramsay Sedation Scale), postoperative shivering (percentage per arm) and pruritus (percentage per arm).

### 2.4. Statistical Analysis

We first conducted a qualitative synthesis of study outcomes. Quantitative synthesis was then performed for outcomes reported by ≥3 studies. Analyses were performed using Cochrane’s Review Manager (RevMan), online version [20]. For continuous outcomes (primary outcomes), pooled mean differences (MDs) based on least square means were calculated using an inverse-variance random-effects model. For dichotomous secondary outcomes, effect estimates were expressed as risk ratios (RRs), with all results presented alongside 95% confidence intervals (CIs). Heterogeneity across trials was evaluated using the Cochrane Q test and quantified with I^2^ (≥75% indicating substantial heterogeneity [21]. Subgroup analysis was performed based on the nalbuphine dose to investigate potential sources of heterogeneity. Sensitivity analysis was conducted based on risk of bias assessment [22]. Publication bias for the primary outcomes was assessed visually using funnel plots although limitations of all methods are considerable when the number of studies is relatively small [23].

### 2.5. Risk of Bias and Certainty of Evidence Assessment

Risk of bias was assessed for each study by two reviewers independently, with a third reviewer resolving any disagreements. The assessment was conducted using the Cochrane Risk of Bias 2 (RoB 2) tool for randomised controlled trials [24]. The primary outcomes were evaluated in relation to randomisation process, deviations from the intended interventions, missing outcome data, measurement of outcome, and selection of the reported result. Based on the overall assessment, studies were categorised as having “low risk”, “some concerns”, or “high risk” of bias. The certainty of the evidence for each outcome was evaluated using the Grading of Recommendations, Assessment, Development, and Evaluation approach implemented through GRADEpro software (GRADEpro, gradepro.org) [25]. This framework considers five key domains to determine the overall certainty: study design, risk of bias, inconsistency, indirectness, and imprecision. Based on this assessment, the certainty of evidence was rated as high, moderate, low, or very low, for all evaluated outcomes, including both primary and secondary.

## 3. Results

### 3.1. Study Selection

**Figure 1:**
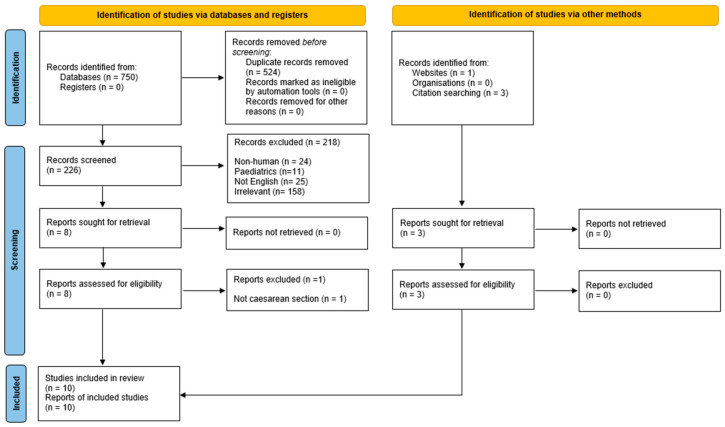
PRISMA 2020 flow diagram for updated systematic reviews which included search of databases, registers and other sources.

A total of 750 records were initially identified through database searches. Following the removal of 524 duplicates, 226 records were retained for screening. During the title and abstract screening process, 218 records were excluded; hence, eight reports were sought for retrieval and subsequently assessed for eligibility [26,27,28,29,30,31,32,33]. One report was excluded as it did not pertain to caesarean section [29]. In addition, three additional references were identified as eligible in the reference lists of the seven relevant articles [34,35,36]. Ultimately, ten studies met the inclusion criteria and were therefore included in the final review [26,27,28,30,31,32,33,34,35,36]. The search strategy allowed for the inclusion of prospective randomised and non-randomised controlled trials, as well as retrospective studies. Coincidentally, all studies meeting the eligibility criteria were classified as randomised controlled trials (RCTs). The process of the study selection is summarised in Figure 1.

### 3.2. Study Characteristics

Six out of the ten studies were conducted in Egypt [26,30,31,33,34,35], three in India [27,32,36] and one in Switzerland [28]. Moreover, five studies were published in the last five years (2021–2025) [26,31,32,33,36], four studies between 2014 and 2019 [27,30,34,35] and only one study was published in 2000 [28]. The number of participants in the included studies varied, with the smallest study examining 60 participants [30] and the largest including 150 participants [27,31,36], for a total of 1095 parturients across all studies. The main characteristics of the included studies are summarised in Table 1.

Administered doses of hyperbaric bupivacaine as the local anaesthetic of choice ranged from 8 mg to 12.5 mg. In one study, the dose of bupivacaine was adjusted according to the height and weight of the parturient [33]. Each study had one arm where nalbuphine was administered with doses ranging from 0.2 mg to 2 mg among the studies. The most commonly used dose was 0.8 mg. Fentanyl was the most common comparator, featured in eight studies [26,27,30,32,33,34,35,36], in five of which it was administered at a dose of 25 mcg [26,30,33,34,35]. The remaining three studies used 20 mcg of fentanyl [27,32,36]. Morphine was used as the comparator in two studies, with doses of 150 mcg and 200 mcg, respectively [28,31]. Additionally, four studies included a normal saline 0.9% arm as a placebo control [26,27,35,36]. The volume of saline administered was adjusted to ensure that all treatment arms received the same total mixture volume. Only one study included an additional arm with midazolam, which is reported solely for reasons of data transparency [26] (Table 1).

In all included studies, a 25 G Quincke needle was used for the administration of spinal anaesthesia, mainly at L3–L4 interspace. Subsequent to anaesthesia administration, parturients were positioned supine and a wedge was applied to achieve left uterine displacement. Mean duration of caesarean section was reported in six studies and ranged from 52 min [32] to 74 min [27] in the nalbuphine group. None of the included studies reported a statistically significant difference in surgical duration among the comparable groups as presented in Table 2.

### 3.3. Demographics

Mean age of parturients in the nalbuphine arms ranged from 24.1 years [36] to 30.2 years [34]. No study reported a statistically significant difference among groups, neither in maternal age nor in ASA physical status score. In the nalbuphine arms, patients’ mean weight ranged from 71.4 kg [26] to 81.5 kg [30], and mean height ranged from 164 cm [34] to 171 cm [33]. No statistically significant differences, neither in weight nor in height, were found between the study arms. Table 3 summarises the demographic details of included studies.

### 3.4. Systematic Review and Meta-Analysis

Meta-analysis was performed only for studies comparing intrathecal nalbuphine with fentanyl [26,27,30,32,33,34,35,36]. Studies comparing nalbuphine with morphine (n = 2) were included in the qualitative synthesis only, due to the insufficient number of studies for reliable quantitative pooling [28,31]. Furthermore, study arms involving comparators other than opioids, such as non-opioid adjuvants or normal saline, were excluded from both the systematic review and meta-analysis, as they were not aligned with the primary objective of this study.

### 3.5. Primary Outcomes

**Figure 2:**
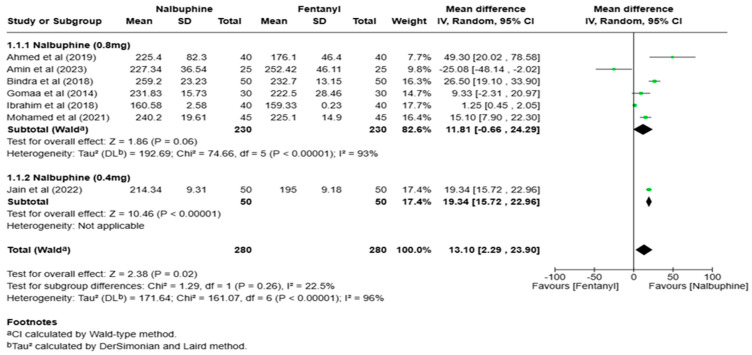
Forest plot of duration of effective analgesia comparing nalbuphine (0.4 mg or 0.8 mg) and fentanyl (20 mcg or 25 mcg); total and subgroup analyses [26,27,30,32,33,34,35].

**Figure 3:**
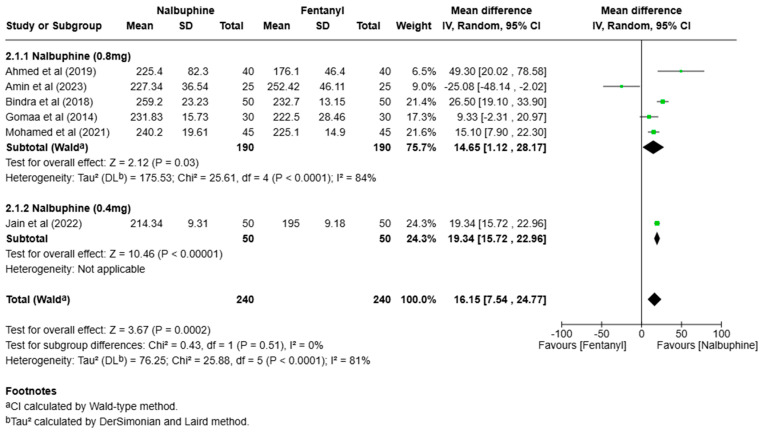
Sensitivity analysis of studies examining duration of effective analgesia in nalbuphine (0.4 mg or 0.8 mg) versus fentanyl (20 mcg or 25 mcg) [26,27,30,32,33,34].

**Figure 4:**
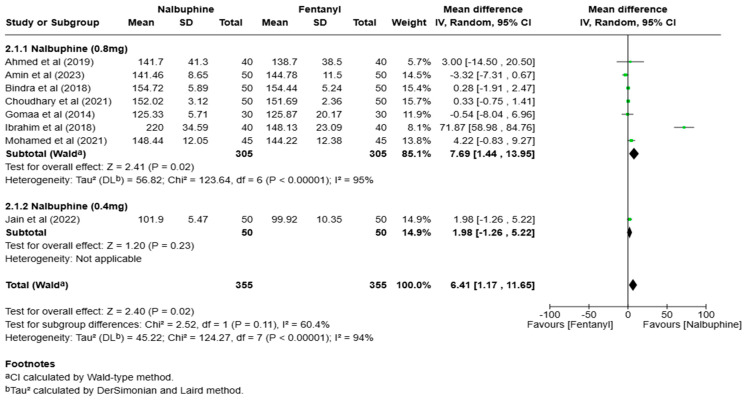
Forest plot of duration of motor block comparing nalbuphine (0.4 mg or 0.8 mg) and fentanyl (20 mcg or 25 mcg); total and subgroup analyses [26,27,30,32,33,34,35,36].

**Figure 5:**
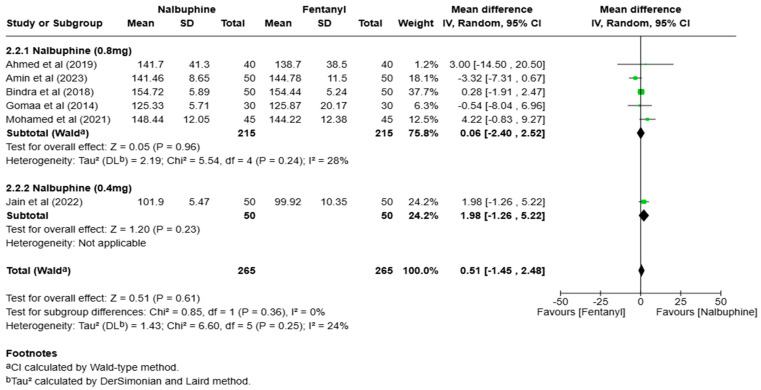
Sensitivity analysis of studies examining duration of motor block in nalbuphine (0.4 mg or 0.8 mg) versus fentanyl (20 mcg or 25 mcg) [26,27,30,32,33,34].

**Figure 6:**
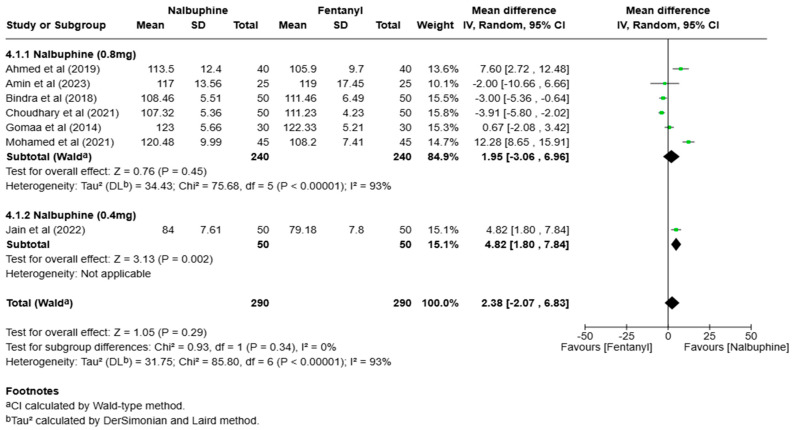
Forest plot of duration of sensory block comparing nalbuphine (0.4 mg or 0.8 mg) and fentanyl (20 mcg or 25 mcg); total and subgroup analyses [26,27,30,32,33,34,36].

**Figure 7:**
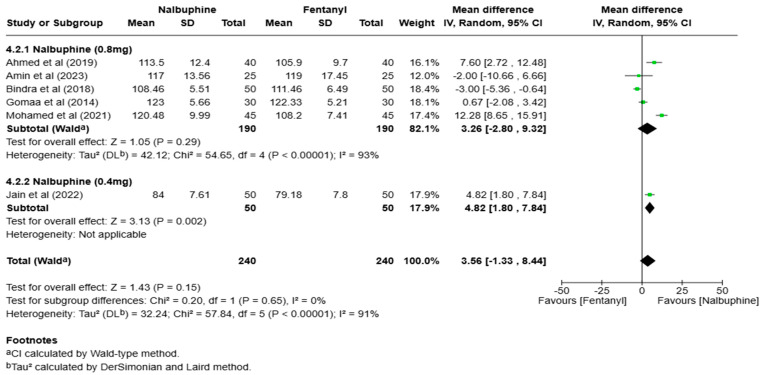
Sensitivity analysis of studies examining duration of sensory block in nalbuphine (0.4 mg or 0.8 mg) versus fentanyl (20 mcg or 25 mcg) [26,27,30,32,33,34].

The primary objective of this systematic review was to compare the duration of effective analgesia, defined as the time interval in minutes (min) from the administration of spinal anaesthesia to the parturients’ first request for rescue analgesia, following caesarean delivery. Seven randomised controlled trials demonstrated a statistically significant prolongation of effective analgesia with nalbuphine compared to fentanyl (MD = 13.10 min, 95% CI 2.29–23.90, *p* = 0.02) (Figure 2), with substantial heterogeneity (I^2^ = 96%) [26,27,30,32,33,34,35]. Subgroup analysis showed no significant difference for the 0.8 mg dose (MD = 11.81 min, 95% CI −0.66 to 24.29, *p* = 0.06; I^2^ = 93%), while the 0.4 mg subgroup demonstrated a significant effect (MD = 19.34 min, 95% CI 15.72–22.96, *p* < 0.00001), without significant subgroup differences (*p* = 0.26) (Figure 2). Sensitivity analysis confirmed the effect (MD = 16.15 min, 95% CI 7.54–24.77, *p* = 0.0002), while heterogeneity decreased but remained substantial (I^2^ = 81%) (Figure 3). For duration of motor block, seven trials showed a statistically significant prolongation with nalbuphine compared to fentanyl (MD = 6.41 min, 95% CI 1.17–11.65, *p* = 0.02; I^2^ = 94%), which was significant in the 0.8 mg subgroup (MD = 7.69 min, 95% CI 1.44–13.95, *p* = 0.02; I^2^ = 95%) but not in the 0.4 mg subgroup (*p* = 0.23) [26,27,30,32,33,34,35,36] (Figure 4). No subgroup differences were observed (*p* = 0.11). However, sensitivity analysis resulted in loss of statistical significance (MD = 0.51 min, 95% CI −1.45 to 2.48, *p* = 0.61) and markedly reduced heterogeneity (I^2^ = 24%) (Figure 5). For the duration of sensory block, no statistically significant difference was observed between nalbuphine and fentanyl groups (MD = 2.38 min, 95% CI −2.07 to 6.83, *p* = 0.29; I^2^ = 93%) (Figure 6) with similar findings after sensitivity analysis (MD = 3.56 min, 95% CI −1.33 to 8.44, *p* = 0.15, I^2^ = 91%) (Figure 7) [26,27,30,32,33,34,36]. Subgroup analysis showed no significant effect for the 0.8 mg dose (MD = 1.95 min, 95% CI −3.06 to 6.96, *p* = 0.45; I^2^ = 93%), whereas the 0.4 mg subgroup, based on a single study, demonstrated a statistically significant prolongation (MD = 4.82 min, 95% CI 1.80–7.84, *p* = 0.002) (Figure 6).

Regarding comparison with morphine, both studies reported that duration of effective analgesia was statistically longer in morphine groups compared to nalbuphine groups. (*p* <0.001—Hassan et al. and *p* < 0.0001—Culebras et al.) [28,31]. Moreover, Hassan et al. reported a statistically significant prolongation of motor block in the morphine group compared to the nalbuphine group (*p* < 0.001) [31]. However, no data were provided for the duration of sensory block, precluding intergroup comparison for this outcome. Primary outcomes of the studies involving morphine are presented collectively in Table 4.

### 3.6. Secondary Outcomes

#### Onset Time of Sensory and Motor Block, and Complete Analgesia

**Figure 8:**
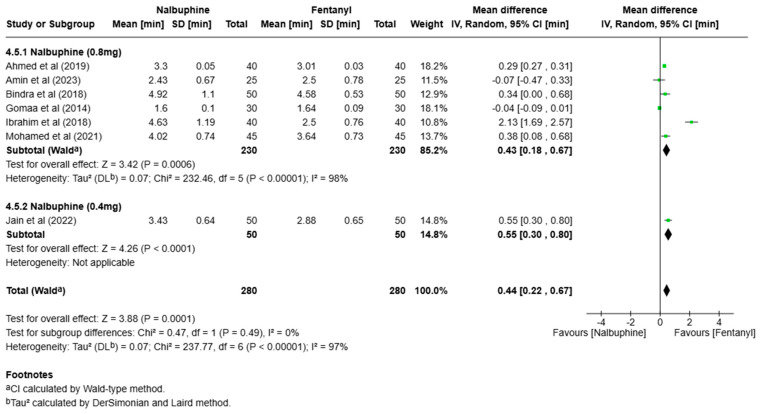
Forest plot of onset of sensory block comparing nalbuphine (0.4 mg or 0.8 mg) and fentanyl (20 mcg or 25 mcg); total and subgroup analyses [26,27,30,32,33,34,35].

**Figure 9:**
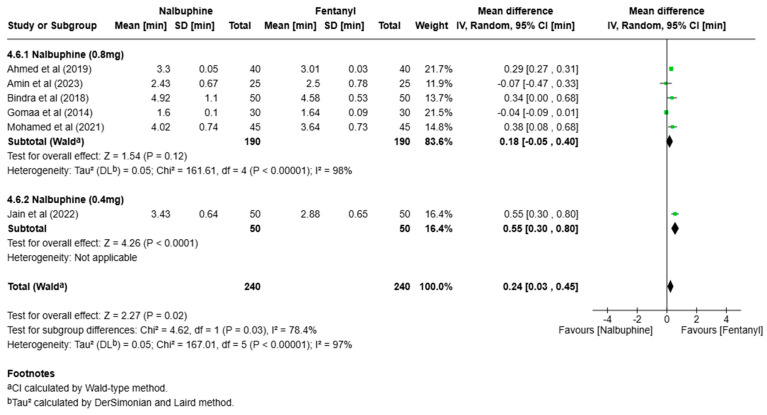
Sensitivity analysis of studies examining onset of sensory block in nalbuphine (0.4 mg or 0.8 mg) versus fentanyl (20 mcg or 25 mcg) [26,27,30,32,33,34].

**Figure 10:**
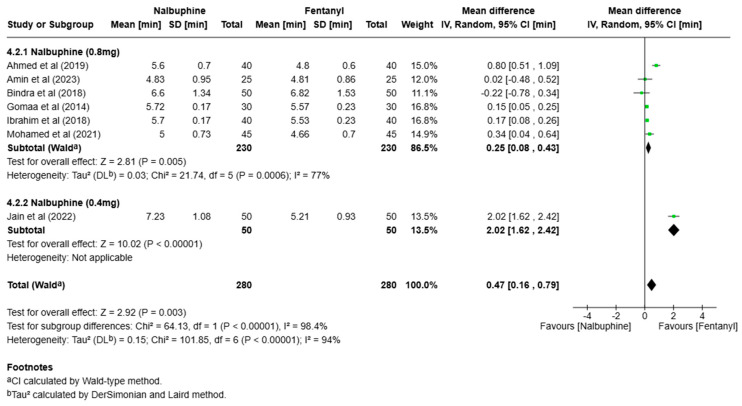
Forest plot of onset of motor block comparing nalbuphine (0.4 mg or 0.8 mg) and fentanyl (20 mcg or 25 mcg); total and subgroup analyses [26,27,30,32,33,34,35].

**Figure 11:**
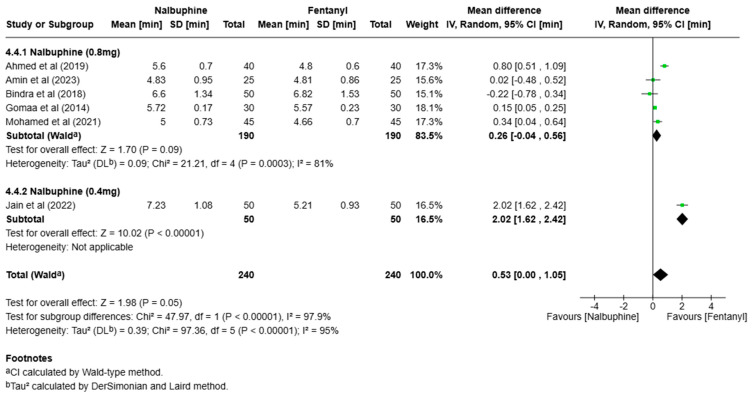
Sensitivity analysis of studies examining onset of motor block in nalbuphine (0.4 mg or 0.8 mg) versus fentanyl (20 mcg or 25 mcg) [26,27,30,32,33,34].

**Figure 12:**
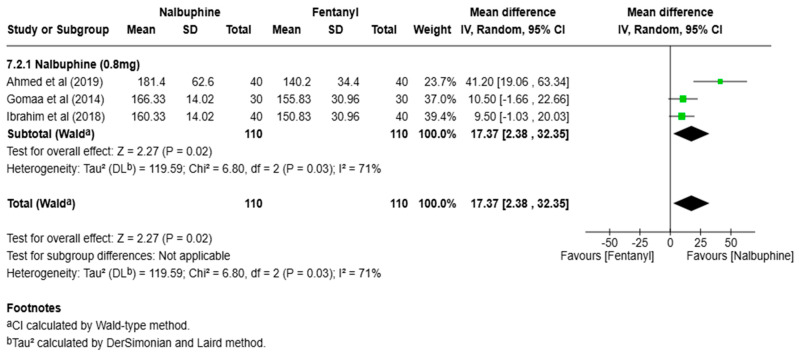
Forest plot of duration of complete analgesia comparing nalbuphine (0.4 mg or 0.8 mg) and fentanyl (20 mcg or 25 mcg) [32,34,35].

Seven trials evaluating onset of sensory block demonstrated a statistically significant delay with nalbuphine (MD = 0.44 min, 95% CI 0.22–0.67, *p* < 0.001; I^2^ = 97%), with consistent findings in the 0.8 mg subgroup (MD = 0.43 min, 95% CI 0.18–0.67, *p* = 0.0006; I^2^ = 98%) and a significant effect in the 0.4 mg subgroup (MD = 0.55 min, 95% CI 0.30–0.80) [26,27,30,32,33,34,35] (Figure 8). No significant subgroup differences were detected (*p* = 0.49). Sensitivity analysis confirmed the effect with reduced magnitude (MD = 0.24 min, 95% CI 0.03–0.45, *p* = 0.02), although heterogeneity remained high (I^2^ = 78.4%) (Figure 9). Notably, after sensitivity analysis, the effect in the 0.8 mg subgroup was no longer statistically significant, whereas the 0.4 mg subgroup remained unchanged, and a significant subgroup difference emerged (*p* = 0.03). It should be noted that, although the predefined sensory block level was T5 across studies, two of the included studies contributing to the forest plot reported a sensory block at T6. Seven trials assessing onset of motor block demonstrated a statistically significant delay with nalbuphine compared to fentanyl. (MD = 0.47 min, 95% CI 0.16–0.79, *p* = 0.003; I^2^ = 94%). Subgroup analysis revealed a modest but statistically significant delay in the 0.8 mg subgroup (MD = 0.25 min, 95% CI 0.08–0.43, *p* = 0,005; I^2^ = 77%) and a larger effect in the 0.4 mg subgroup (MD = 2.02 min, 95% CI 1.62–2.42, *p* < 0.00001) [26,27,30,32,33,34,35] (Figure 10). However, the overall heterogeneity was considerable, suggesting important variability among the included studies. Sensitivity analysis resulted in a borderline non-significant pooled effect (MD = 0.53 min, 95% CI 0.00–1.05, *p* = 0.05; I^2^ = 95%) (Figure 11), suggesting that the primary finding lacks robustness and remains affected by considerable heterogeneity. Lastly, three studies evaluating the duration of complete analgesia (time until VAS > 0) demonstrated a statistically significant prolongation in the nalbuphine group compared to fentanyl (MD = 17.37 min, 95% CI 2.38–32.35, *p* = 0.02) [32,34,35]. However, this effect was accompanied by moderate heterogeneity (I^2^ = 71%). While one study showed a more pronounced benefit, the remaining studies reported smaller and non-significant differences, contributing to the observed variability (Figure 12). 

Regarding morphine studies, they did not report onset time of sensory and motor block [28,31]. Furthermore, only Culebras et al. reported the duration of complete analgesia, demonstrating that morphine significantly prolonged complete analgesia compared to all nalbuphine doses (*p* < 0.0001) [28]. All secondary outcomes of the studies involving morphine are presented in Table 5.

### 3.7. Adverse Effects

**Figure 13:**
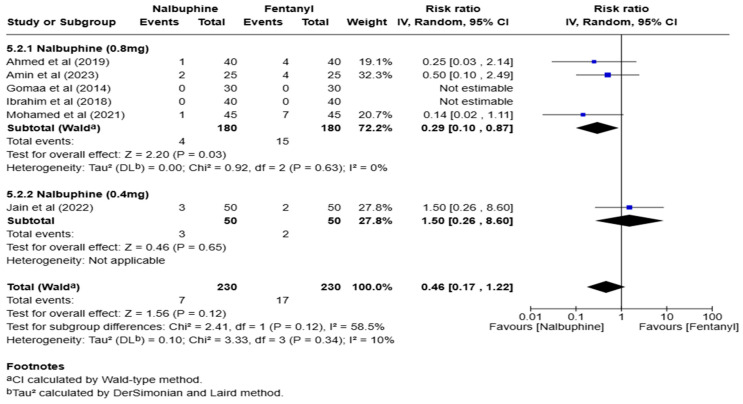
Forest plot of bradycardia events comparing nalbuphine (0.4 mg or 0.8 mg) and fentanyl (20 mcg or 25 mcg); total and subgroup analyses [26,30,32,33,34,35].

**Figure 14:**
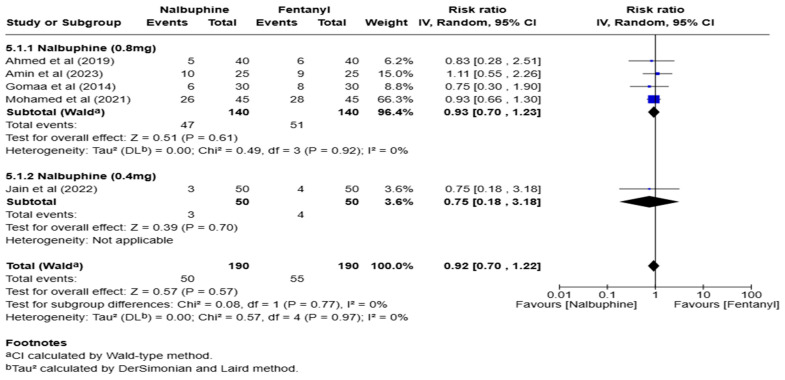
Forest plot of hypotension events comparing nalbuphine (0.4 mg or 0.8 mg) and fentanyl (20 mcg or 25 mcg); total and subgroup analyses [26,30,32,33,34].

**Figure 15:**
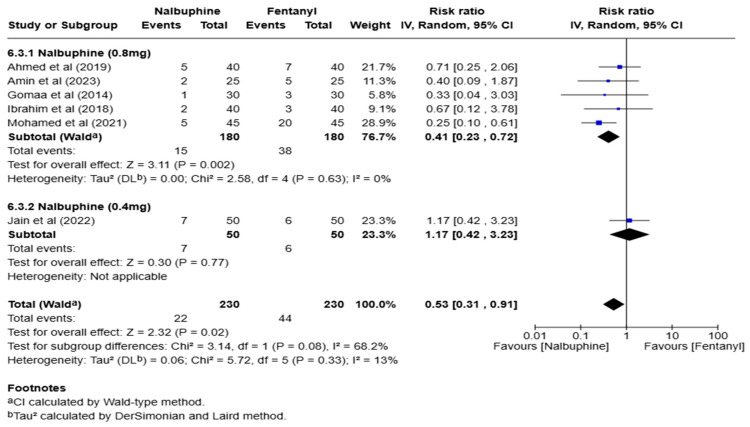
Forest plot of PONV events comparing nalbuphine (0.4 mg or 0.8 mg) and fentanyl (20 mcg or 25 mcg); total and subgroup analyses [26,30,32,33,34,35].

**Figure 16:**
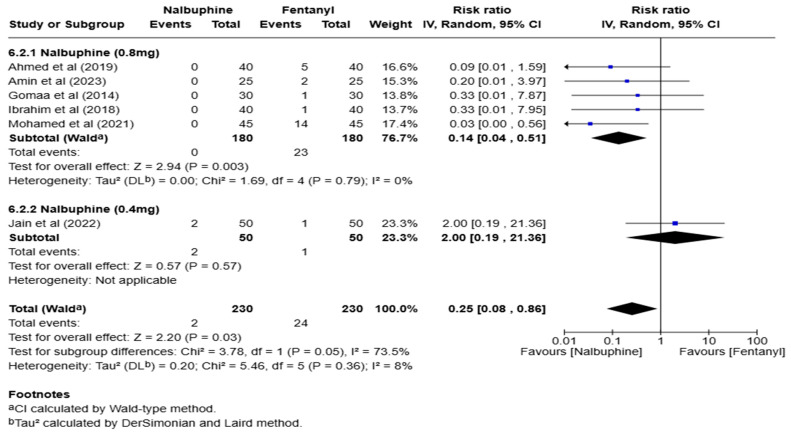
Forest plot of pruritus events comparing nalbuphine (0.4 mg or 0.8 mg) and fentanyl (20 mcg or 25 mcg); total and subgroup analyses [26,30,32,33,34,35].

**Figure 17:**
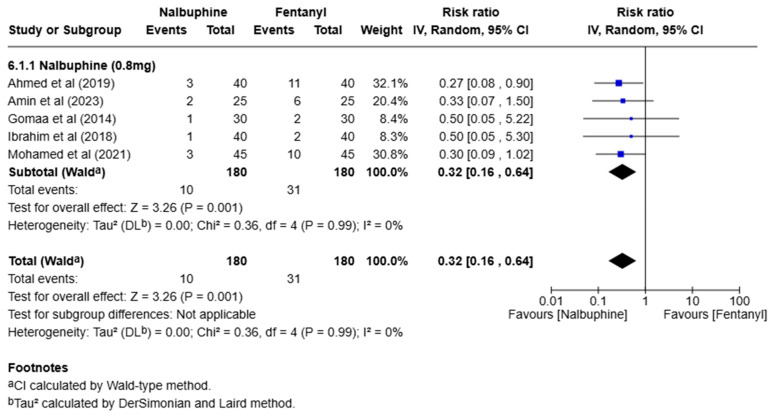
Forest plot of shivering events comparing nalbuphine (0.4 mg or 0.8 mg) and fentanyl (20 mcg or 25 mcg) [26,30,33,34,35].

**Figure 18:**
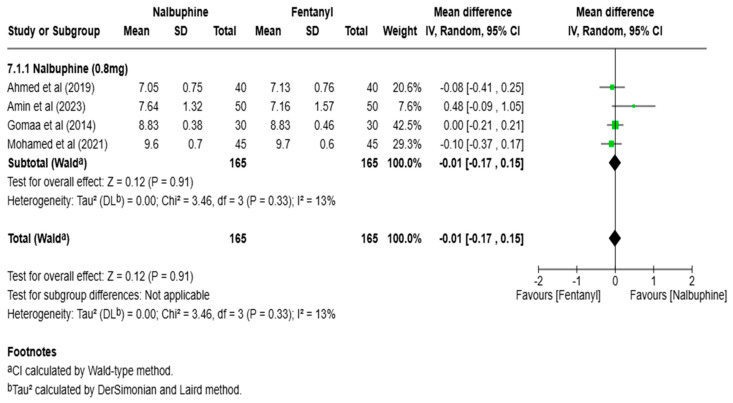
Forest plot of Apgar score at 1 min comparing nalbuphine (0.8 mg) and fentanyl (25 µg) [26,30,33,34].

**Figure 19:**
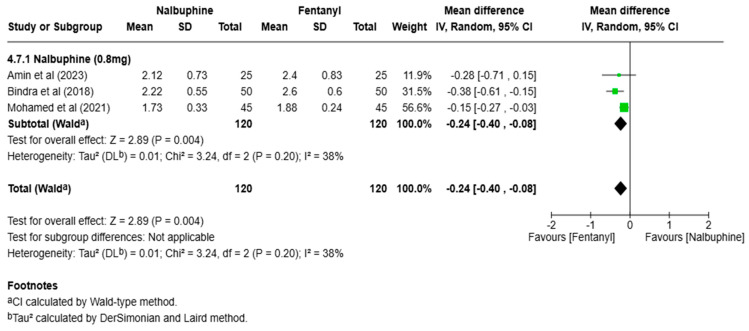
Forest plot of studies examining Sedation (RSS) in nalbuphine (0.4 mg or 0.8 mg) versus fentanyl (20 mcg or 25 mcg) [26,27,33].

Analysis of adverse outcomes showed no statistically significant differences between nalbuphine and fentanyl for bradycardia (RR = 0.46, 95% CI 0.17–1.22, *p* = 0.12; I^2^ = 10%), although a trend toward lower incidence with nalbuphine was noted [26,30,32,33,34,35] (Figure 13). Subgroup analysis, however, revealed a statistically significant reduction in bradycardia incidence with nalbuphine at a dose of 0.8 mg (RR: 0.29, 95% CI: 0.10 to 0.87; *p* = 0.03). Regarding hypotension, pooled analysis revealed no statistically significant difference between groups (RR = 0.92, 95% CI 0.70–1.22, *p* = 0.57, I^2^ = 0%) [26,30,32,33,34]. (Figure 14). Subgroup analyses yielded consistent findings. Remarkably, nalbuphine was associated with a statistically significant reduction in PONV (RR = 0.53, 95% CI 0.31–0.91, *p* = 0.02; I^2^ = 13%) [26,30,32,33,34,35] (Figure 15) and pruritus (RR = 0.25, 95% CI 0.08–0.86, *p* = 0.03; I^2^ = 8%) [26,30,32,33,34,35] (Figure 16), particularly at the 0.8 mg dose. Additionally, five trials demonstrated a significant reduction in shivering with nalbuphine (RR = 0.32, 95% CI 0.16–0.64, *p* = 0.001; I^2^ = 0%), corresponding to an absolute reduction of 117 cases per 1000 patients compared with fentanyl [26,30,33,34,35] (Figure 17). Neonatal outcomes, assessed by Apgar score at 1 min, were comparable between groups (MD = −0.01, 95% CI −0.17 to 0.15, *p* = 0.91; I^2^ = 13%) [26,30,33,34] (Figure 18). Only two studies reported Apgar scores at 5 min, and no statistically significant difference was observed [26,34]. Regarding respiratory depression, five studies reported no cases in either the nalbuphine or fentanyl groups [26,28,33,34,35]. Furthermore, intrathecal nalbuphine was associated with slightly lower sedation scores compared to fentanyl (MD −0.24, 95% CI −0.40 to −0.08), with low heterogeneity across studies (I^2^ = 38%) (Figure 19). Notably, four studies reported sedation outcomes; however, meta-analysis was performed on the three studies [26,27,33] that assessed sedation using the Ramsay Sedation Scale (RSS). The fourth study [34] reported sedation as event counts and was therefore not included in the quantitative synthesis.

When assessing adverse events in comparisons between nalbuphine and morphine, data were available for a limited number of outcomes. Both included studies reported a significantly lower incidence of pruritus in the nalbuphine group compared to morphine, irrespective of nalbuphine dose (Hassan et al., *p* = 0.018; Culebras et al., *p* = 0.0001) [28,31]. For postoperative nausea and vomiting (PONV), only Culebras et al. demonstrated a statistically significant reduction in the nalbuphine group (*p* < 0.05) [28], whereas Hassan et al. reported no significant difference (*p* = 0.131) [31]. Additionally, Hassan et al. found no statistically significant difference in the incidence of bradycardia between groups, although no numerical data were provided [31]. All adverse effects reported in the studies involving morphine are summarised in Table 5 for comparison.

### 3.8. Risk of Bias and Certainty of Evidence Assessment

**Figure 20:**
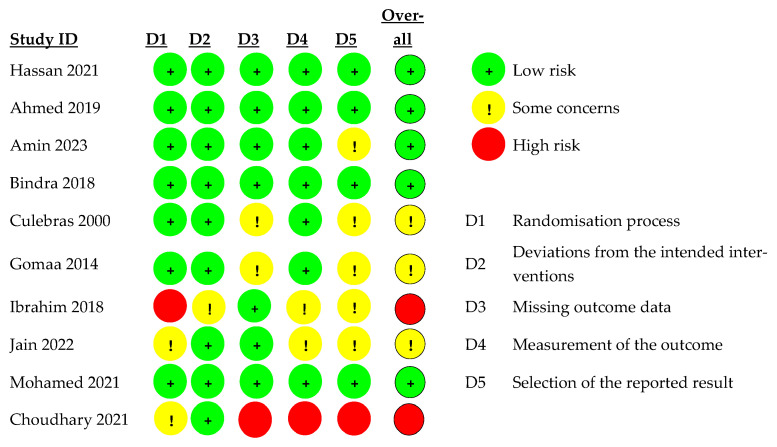
RoB2 criteria matrix [27,28,29,31,32,33,34,35,36,37].

Risk of Bias 2 (RoB 2) tool was applied as summarised in Figure 20. Five studies were assessed as having a low risk of bias [26,27,31,33,34], while three were found to have “some concerns” [28,30,32]. The study by Ibrahim et al. was considered to have a high risk of bias due to insufficient reporting of the randomisation process, and lack of blinding [35]. The study by Choudhary et al. was rated as high risk of bias due to missing data, lack of reporting of statistical methods, and shortcomings in blinding and randomisation [36]. The most frequently identified source of bias was domain D5 (bias in selection of the reported result), primarily due to absence of a pre-specified analysis plan in a publicly accessible registry such as ClinicalTrials.gov.

The certainty of evidence varied across outcomes according to the GRADE approach. The corresponding GRADE summary table is provided in the Supplementary Material. High-certainty evidence was found for sedation, indicating that intrathecal nalbuphine is associated with slightly lower sedation scores compared to fentanyl. Similarly, high-certainty evidence for Apgar score at 1 min suggests no clinically relevant differences in neonatal condition. Moderate-certainty evidence supported reductions in shivering and postoperative nausea and vomiting (PONV), whereas no significant difference was found for hypotension. In contrast, the certainty of evidence was low for onset of motor and sensory block and for bradycardia, mainly due to concerns regarding risk of bias and inconsistency. Evidence for duration of effective and complete analgesia was rated as very low, primarily due to substantial heterogeneity and imprecision. Similarly, the certainty of evidence for pruritus was very low, despite a reduction in events, owing to serious concerns related to risk of bias, inconsistency, and imprecision. Overall, while some outcomes suggest potential advantages of nalbuphine over fentanyl, the confidence in these estimates is limited for several endpoints. Further well-designed, adequately powered randomised controlled trials are required to provide more robust and reliable evidence.

### 3.9. Publication Bias

**Figure 21:**
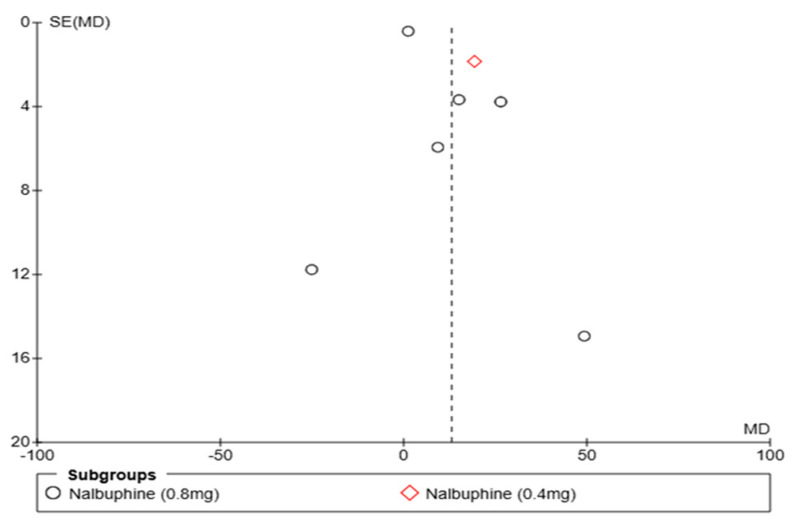
Funnel plot for duration of effective analgesia in nalbuphine (0.4 mg or 0.8 mg) versus fentanyl (20 mcg or 25 mcg) [26,27,30,32,33,34,35].

**Figure 22:**
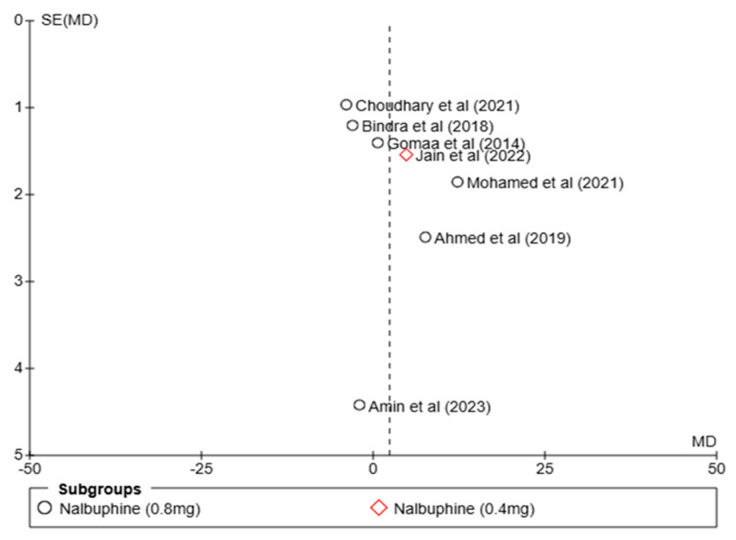
Funnel plot for duration of sensory block in nalbuphine (0.4 mg or 0.8 mg) versus fentanyl (20 mcg or 25 mcg) [26,27,30,32,33,34,36].

**Figure 23:**
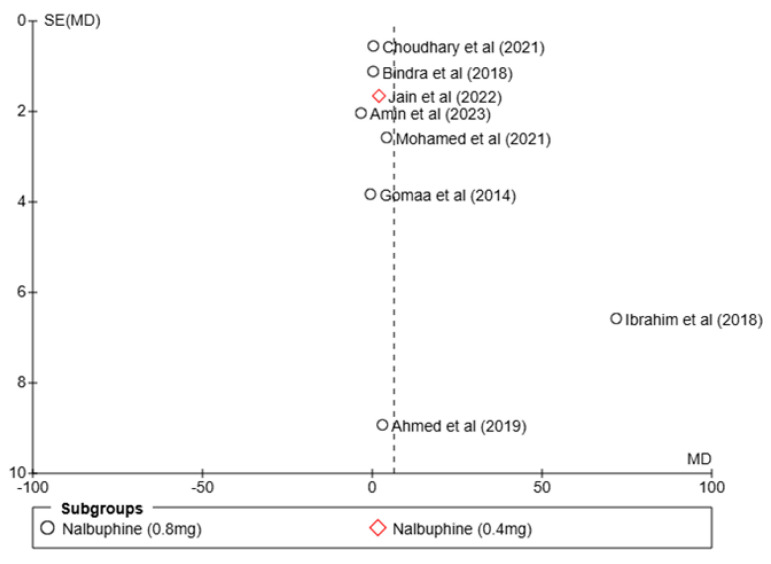
Funnel plot for duration of motor block in nalbuphine (0.4 mg or 0.8 mg) versus fentanyl (20 mcg or 25 mcg), suggesting possible publication bias [26,27,30,32,33,34,35,36].

Publication bias was assessed visually using funnel plots for all outcomes. Overall, the funnel plots appeared largely symmetrical, suggesting no clear evidence of publication bias. Funnel plots for the duration of effective analgesia and duration of sensory block are presented in Figure 21 and Figure 22, respectively. However, for the duration of motor block, the funnel plot showed some asymmetry (Figure 23). This may suggest the presence of publication bias, particularly as smaller studies with negative or null effects may be missing. Nevertheless, these findings should be interpreted with caution given the limited number of included studies for most outcomes, which restricts the reliability of funnel plot assessment. Funnel plots for secondary outcomes are presented in the Supplementary Material.

## 4. Discussion

This systematic review evaluated the effectiveness and safety of intrathecal nalbuphine versus other opioid adjuvants to hyperbaric bupivacaine in anaesthesia for caesarean section. The meta-analysis of nalbuphine versus fentanyl suggests that nalbuphine may be associated with a statistically significant prolongation of effective analgesia and motor block duration compared to fentanyl. However, these differences are modest and unlikely to be clinically meaningful. They should be interpreted with caution given the very low certainty of evidence, which is primarily driven by concerns related to risk of bias, substantial heterogeneity across studies, and imprecision of the effect estimates. For motor block duration, potential publication bias may further limit confidence in the findings. The observed prolongation of analgesia by approximately 10–15 min and motor block by only a few minutes would not substantially impact clinical practice or patient management. Furthermore, the inconsistency across subgroups, particularly the lack of significance in the 0.8 mg dose and the reliance on single-study findings for the 0.4 mg dose, limits conclusions regarding a dose–response relationship. The loss of statistical significance in motor block duration after sensitivity analysis highlights the influence of study quality and suggests that this finding may not be robust. In contrast, the duration of sensory block was comparable between nalbuphine and fentanyl, further supporting the overall similarity in anaesthetic profile between the two agents. The high heterogeneity observed across most analyses likely reflects differences in study design, anaesthetic techniques, and outcome definitions. Overall, these findings may indicate that nalbuphine provides an anaesthetic profile comparable to fentanyl with respect to block characteristics, with only minimal differences that are unlikely to be clinically relevant. On the contrary, Naaz et al. demonstrated statistically and clinically significant superiority of intrathecal nalbuphine over fentanyl as an adjuvant to bupivacaine in lower limb surgeries [37]. The duration of effective analgesia in the nalbuphine group was extended (mean difference of 145.5 min). Statistically and clinically significant superiority of nalbuphine was also reported by Gupta et al. in orthopaedic cases [38]. In this study, duration of analgesia was also longer in the nalbuphine group compared to the fentanyl group (mean difference of 144 min). As for the regression time of sensory and motor block, Deori et al. reported a significantly longer duration with nalbuphine 0.8 mg compared to fentanyl 25 mcg in lower abdomen surgeries [29].

According to the meta-analysis findings, nalbuphine was associated with a statistically significant delay in both sensory and motor block onset. However, given that the magnitude of these differences was very small—generally less than one minute—and the certainty of evidence was low, they are unlikely to be clinically meaningful. This low certainty is primarily driven by concerns related to risk of bias and inconsistency across studies. Although statistical significance was achieved in the primary analyses, the high heterogeneity, the loss of significance in sensitivity analyses and the low certainty of evidence suggest that these findings should be interpreted with caution. Overall, the minimal delay in onset is unlikely to influence clinical decision-making and does not represent a disadvantage in routine practice. In contrast, nalbuphine demonstrated a statistically significant prolongation in the duration of complete analgesia compared to fentanyl. However, the magnitude of this benefit was modest, and its clinical relevance remains uncertain. Importantly, the certainty of evidence was very low, mainly due to risk of bias, inconsistency across studies, and imprecision. Taken together, these findings suggest that while nalbuphine may offer a slight advantage in prolonging analgesia, its overall clinical performance remains broadly comparable to fentanyl.

An essential, consistent finding of this meta-analysis is the more favourable side-effect profile of nalbuphine compared to fentanyl. While no significant differences were observed in haemodynamic parameters such as bradycardia and hypotension, nalbuphine was associated with significant reductions in PONV and shivering (relative reduction of approximately 68%). Low heterogeneity supported the robustness of these findings, which are in accordance with Naaz et al.’s findings [37]. These effects are likely attributable to its pharmacological profile as a κ-opioid receptor agonist and μ-opioid receptor antagonist, which is associated with reduced incidence of μ-receptor-mediated adverse effects. Importantly, neonatal outcomes were comparable between groups, indicating that nalbuphine does not compromise early neonatal adaptation. From a clinical perspective, the reduction in common and distressing side effects represents a meaningful advantage and may improve maternal comfort and overall perioperative experience. However, the limited number of studies in some subgroups and the variability in outcome reporting warrant cautious interpretation, and further high-quality randomised trials are needed to confirm these findings and better define optimal dosing strategies.

As only two studies comparing nalbuphine with morphine were identified, a meta-analysis was not feasible, and the findings were therefore synthesised narratively [28,31]. Specifically, evidence from Culebras et al. and Hassan et al. demonstrated that morphine provides a markedly longer duration of effective analgesia compared to nalbuphine (mean differences of approximately 450 and 350 min, respectively) [28,31]. However, PONV incidence and pruritus incidence were statistically significantly higher in the morphine group. These findings are consistent with those reported by Yoon et al., where the duration of effective analgesia was more than twice as long with intrathecal morphine compared to nalbuphine in caesarean section [39]. Nevertheless, the incidence of pruritus was significantly lower in the nalbuphine group. The effectiveness of morphine in providing robust analgesia was also highlighted by Ollosu et al., who conducted a systematic review and network meta-analysis [40]. This meta-analysis examined the use of all potential adjuncts in caesarean section, without focusing specifically on opioids or comparing them with nalbuphine. They also reported that the duration of effective analgesia is significantly prolonged when morphine is combined with other agents, such as neostigmine or nalbuphine.

Although not within the primary scope of this review, data comparing nalbuphine and fentanyl with normal saline control groups were also extracted and reported to enhance transparency. Across studies including a control arm, both opioids consistently demonstrated superiority over saline, with significantly longer duration of effective analgesia [26,27,33], motor block [26,27,33,36], and sensory block [26,27,33], as well as faster onset of sensory block [26,33]. Interestingly, higher incidences of nausea, vomiting, and shivering were observed in the normal saline group in one study, but authors did not provide an explanation for this finding [33]. No differences were identified in hypotension. Sedation scores were lower in the control groups compared to both opioid groups [26,33]. Collectively, these findings support the well-established benefit of intrathecal opioid co-administration with local anaesthetics over local anaesthetic alone. These observations are in line with previous studies, such as that by Tiwari et al., who demonstrated that intrathecal nalbuphine (0.4 mg) significantly prolonged sensory block and postoperative analgesia without increasing adverse effects compared to bupivacaine alone [41]. Furthermore, evidence from non-obstetric settings suggests a potential ceiling effect of nalbuphine, whereby increasing doses do not result in further analgesic benefit. This is supported by findings from Jyothi et al., as well as by the present review, where no significant difference was observed between intermediate and higher nalbuphine doses [42]. Specifically, the duration of effective analgesia was statistically significant longer in Culebras et al.’s study, comparing 0.8 mg to 0.2 mg nalbuphine groups, and 1.6 mg to 0.2 mg nalbuphine groups, but not between 0.8 mg and 1.6 mg groups [28]. No difference was found either in the study by Hassan et al., when nalbuphine groups of 1 mg and 2 mg were compared [31]. Notably, higher nalbuphine doses were associated with increased incidence of PONV and pruritus [28].

In this systematic review and meta-analysis, no study comparing nalbuphine to intrathecal opioids other than fentanyl and morphine in caesarean section was retrieved. However, in other types of surgery, nalbuphine has been compared with additional intrathecal opioid adjuvants. In orthopaedic surgeries, Elsayed et al. found that nalbuphine 0.5 mg juxtaposed with pethidine 10 mg intrathecally provided a comparable duration of effective analgesia [43]. Sundaram et al. reported that nalbuphine, compared to tramadol in the subarachnoid space, offered longer analgesia with comparable onset and safety in infraumbilical surgeries [44].

Lastly, regarding study quality, two studies were assessed as having a high risk of bias. However, they did not demonstrate statistically significant differences between groups. Sensitivity analyses excluding these studies resulted in changes in some pooled estimates, while most remained unchanged, suggesting a partial influence on the results without substantially altering the overall conclusions [35,36].

Nalbuphine, as a mixed opioid agonist–antagonist, appears to be associated with a lower incidence of common opioid-related adverse effects, particularly pruritus, shivering, and postoperative nausea and vomiting (PONV). As widely established in the literature, intrathecal morphine remains the gold standard for obstetric analgesia mainly because of prolonged postpartum analgesia [10]. However, in cases where patients have previously experienced significant opioid-related side effects—either following caesarean section or other procedures involving intrathecal morphine or fentanyl—nalbuphine may represent a reasonable alternative. Evidence from the included studies and from the literature suggests that nalbuphine consistently provides superior analgesia compared with no opioid use [45]. At the same time, it demonstrates a duration of analgesia comparable to fentanyl. Taken together, these findings support the potential role of nalbuphine as an alternative intrathecal opioid in selected patients, particularly when tolerability is a primary concern.

### Limitations

Despite offering valuable insights, this systematic review and meta-analysis is constrained by several limitations stemming from the included studies. Most included studies were characterised by small sample sizes and geographical clustering, primarily in Egypt and India. This geographical trend may be attributed to the drug’s availability and low cost. Consequently, the lack of data from Western cohorts or multi-continental trials may limit the extrapolation of these results to different healthcare infrastructures. It may also restrict generalisability to diverse ethnic populations with varying genetic or cultural responses to analgesia. The predominance of single-centre designs, coupled with variations in perioperative analgesic protocols and local clinical practices, may limit the external validity of our findings. Furthermore, the absence of formal sample size calculations increases the risk of Type II errors. These limitations, alongside potential publication bias and small-study effects, necessitate a cautious interpretation of the results. Also, assessment of postoperative VAS score was, on most occasions, reported only in charts. The authors mentioned reduced analgesic requirements, but lack detailed numerical data. Therefore, a clear conclusion about nalbuphine’s impact on VAS score cannot be reached. Another notable limitation of the current evidence base is the inconsistent reporting of functional recovery and neonatal outcomes. Urinary retention was evaluated in only one study, a finding potentially confounded by the routine perioperative use of urinary catheters in parturients. Similarly, delayed mobilisation—a key indicator of opioid-related side effects—was entirely unaddressed. Furthermore, while neonatal Apgar scores do not suggest respiratory depression, the available data were limited and the reporting was fragmented. For instance, at the 5 min mark, data were available for only four studies [26,28,31,34]. This lack of standardised outcome measures renders the evidence regarding the intervention’s comprehensive safety profile inconclusive. As a result, its overall clinical impact cannot be clearly determined.

A key limitation of this review is the paucity of studies comparing nalbuphine with intrathecal morphine; only two studies were retrieved [28,31]. Furthermore, these studies did not report an adequate variety of primary and secondary outcomes. This limits the clinical applicability of the findings. Additionally, the relatively small difference observed in analgesia duration between nalbuphine and fentanyl (approximately 15 min) may not translate into meaningful clinical benefit. However, it suggests that nalbuphine may represent a reasonable alternative to fentanyl, with broadly comparable analgesic efficacy.

Finally, although a meta-analysis between nalbuphine and fentanyl was performed, several important limitations should be acknowledged. Many of the included analyses were based on a small number of studies and relatively limited sample sizes, which may reduce statistical power and increase the risk of imprecision. Substantial heterogeneity was observed across multiple outcomes, likely reflecting variability in study design, including differences in nalbuphine dosing (0.2–2 mg), fentanyl dosing (20–25 μg), and the dose of hyperbaric bupivacaine (10–12 mg). Additional heterogeneity may arise from inconsistencies in outcome definitions and measurement approaches across studies. Although subgroup and sensitivity analyses were undertaken to explore these differences, residual heterogeneity remained, which may limit the reliability and interpretability of pooled estimates. Therefore, the results should be interpreted with caution, particularly where high I^2^ values were observed. These methodological limitations are also captured in the GRADE assessment, which demonstrated variability in the certainty of evidence, with several important outcomes rated as low or very low certainty due to risk of bias, heterogeneity, and imprecision. Further well-designed, adequately powered trials with standardised protocols are warranted to confirm these findings.

## 5. Conclusions

Intrathecal nalbuphine as an adjuvant to bupivacaine for caesarean section may provide an analgesic effect broadly comparable to fentanyl. However, it remains inferior to morphine regarding the duration of effective postpartum analgesia. Notably, its potentially superior safety profile—reduced incidence of pruritus, PONV, and shivering—positions it as a valuable clinical alternative, particularly for patients prone to opioid-related side effects. The lack of significant impact on motor and sensory block duration supports its use in obstetric anaesthesia. Ultimately, nalbuphine may represent a balanced choice between efficacy and tolerability. Adequately powered randomised prospective studies, with consistent assessment and thorough reporting of outcomes, are necessary to better define the role of nalbuphine in obstetric clinical practice.

## Figures and Tables

**Table 1:** Main characteristics of included studies.

First Author (Year)		Type of Study	Country of Study	Study Arms (Dose)		Number of Patients
1	Amin et al. (2023) [26]	RCT	Egypt	Nalbuphine (0.8 mg)	Fentanyl (25 mcg)	25 vs. 25 vs. 25 vs. 25
					N/S	
					Midazolam (2 mg)	
2	Bindra et al. (2018) [27]	RCT	India	Nalbuphine (0.8 mg)	Fentanyl (20 mcg)	50 vs. 50 vs. 50
					N/S	
3	Gomaa et al. (2014) [30]	RCT	Egypt	Nalbuphine (0.8 mg)	Fentanyl (25 mcg)	30 vs. 30
4	Mohamed et al. (2021) [33]	RCT	Egypt	Nalbuphine (0.8 mg)	Fentanyl (25 mcg)	45 vs. 45 vs. 45
					N/S	
5	Jain et al. (2022) [32]	RCT	India	Nalbuphine (0.4 mg)	Fentanyl (20 mcg)	50 vs. 50
6	Culebras et al. (2000) [28]	RCT	Switzerland	Nalbuphine (0.2 mg)	Morphine (0.2 mg)	22 vs. 23 vs. 23 vs. 22
				Nalbuphine (0.8 mg)		
				Nalbuphine (1.6 mg)		
7	Hassan et al. (2021) [31]	RCT	Egypt	Nalbuphine (1 mg)	Morphine (0.15 mg)	50 vs. 50 vs. 50
				Nalbuphine (2 mg)		
8	Ahmed et al. (2019) [34]	RCT	Egypt	Nalbuphine (0.8 mg)	Fentanyl (25 mcg)	40 vs. 40
9	Ibrahim et al. (2018) [35]	RCT	Egypt	Nalbuphine (0.8 mg)	Fentanyl (25 mcg)	40 vs. 40
10	Choudhary et al. (2021) [36]	RCT	India	Nalbuphine (0.8 mg)	Fentanyl (20 mcg)	50 vs. 50 vs. 50
					N/S	

RCT: randomised controlled trial, N/S: normal saline.

**Table 2:** Procedural details of included studies.

First Author (Year)		Hyperbaric Bupivacaine Dose (mg)	Segment of Spinal Anaesthesia	Needle Size	Duration of Surgery (min, Mean ± SD)	
					NALBUPHINE	ADJUVANT
1	Amin et al. (2023) [26]	12.5	L3–L4	25 G	56.80 ± 10.02	F: 57.76 ± 11.29
2	Bindra et al. (2018) [27]	10	NR	NR	73.7 ± 21.11	F: 74 ± 21.38
3	Gomaa et al. (2014) [30]	10	L3–4/L4–5	25 G	53.17 ± 4.82	F: 53.00 ± 5.19
4	Mohamed et al. (2021) [33]	DOSE ACCORDING TO W&H	L3–4/L4–5	25 G	NR	NR
5	Jain et al. (2022) [32]	8	L3–L4/L4–L5	25 G	52.24 ± 5.04	F: 51.84 ± 4.94
6	Culebras et al. (2000) [28]	10	L3–L4	27 G	NR	NR
7	Hassan et al. (2021) [31]	10	L3–L4	25 G	NR	NR
8	Ahmed et al. (2019) [34]	12.5	L3–4/L4–5	25 G	56.7 ± 9.6	F: 54.6 ± 10.4
9	Ibrahim et al. (2018) [35]	10	NR	NR	57.17 ± 6.82	F: 57 ± 8.19
10	Choudhary et al. (2021) [36]	10	NR	NR	NR	NR

N: nalbuphine, F: fentanyl, NR: not reported. W&H: weight & height. No statistically significant difference in any comparison.

**Table 3:** Demographic data of included studies.

First Author (Year)		Age (Years, Mean ± SD)		Weight (kg, Mean ± SD)		Height (cm, Mean ± SD)		ASA I–II, I (n, %)	
		NALBUPHINE	ADJUVANT	NALBUPHINE	ADJUVANT	NALBUPHINE	ADJUVANT	NALBUPHINE	ADJUVANT
1	Amin et al. (2023) [26]	26.52 ± 5.38	F: 28.76 ± 4.44	71.40 ± 7.29	F: 69.48 ± 7.05	169.08 ± 4.13	F: 166.88 ± 3.63	II (100%)	
2	Bindra et al. (2018) [27]	26.1 ± 3.92	F: 26.14 ± 4.41	71.88 ± 6.86	F: 72.02 ± 5.63	NR	NR	38 (76%)	F: 36 (72%)
3	Gomaa et al. (2014) [30]	26.97 ± 5.40	F: 26.33 ± 6.08	81.53 ± 9.85	F: 78.83 ± 8.26	170.30 ± 6.94	F: 168.97 ± 5.22	I–II	
4	Mohamed et al. (2021) [33]	29 ± 4.4	F: 28.3 ± 5.3	79.8 ± 8.2	F: 79.9 ± 7.3	170.6 ± 5.8	F: 171.4 ± 4.7	33 (73.3%)	F: 34 (75.6%)
5	Jain et al. (2022) [32]	24.94 ± 1.93	F: 24.98 ± 2.15	NR	NR	NR	NR	40 (80%)	F: 45 (90%)
6	Culebras et al. (2000) [28]	N (0.2 mg): 29 ± 4	M: 26.3 ± 3.47	76 ± 12	M: 81 ± 11	164 ± 5	M: 167 ± 5	I–II	
		N (0.8 mg): 31 ± 5		80 ± 11		164 ± 6			
		N (1.6 mg): 31 ± 5		76 ± 10		165 ± 4			
7	Hassan et al. (2021) [31]	N (1 mg): 26.78 ± 4.05	M: 30 ± 4	N (1 mg): 26.22 ± 2.38 * N (2 mg): 26.31 ± 2.16 *	M: 25.82 ± 2.49 *	NR	NR	N (1 mg): 35 (70%)	M: 32 (64%)
		N (2 mg): 25.52 ± 4.11						N (2 mg): 33 (66%)	
8	Ahmed et al. (2019) [34]	30.2 ± 4.6	F: 31.7 ± 5.3	81.2 ± 11.4	F: 80.4 ± 11.6	164.3 ± 5.8	F: 165.7 ± 4.5	34 (85%)	F: 35 (87.5%)
9	Ibrahim et al. (2018) [35]	26.97 ± 5.40	F: 26.33 ± 6.08	81.53 ± 9.85	78.83 ± 8.26	170.30 ± 6.94	168.97 ± 5.22	I–II	
10	Choudhary et al. (2021) [36]	24.12 ± 3.16	F: 25.21 ± 2.5	NR	NR	NR	NR	31 (77.5%)	F: 30 (75%)

N: nalbuphine, F: fentanyl, M: morphine, NR: not reported, * BMI. No statistically significant difference in any comparison.

**Table 4:** Primary outcomes of studies comparing nalbuphine and morphine.

PRIMARY OUTCOMES	Culebras et al. (2000) [28]				Hassan et al. (2021) [31]		
	NALBUPHINE			MORPHINE	NALBUPHINE		MORPHINE
	0.2 mg	0.8 mg	1.6 mg	0.2 mg	1 mg	2 mg	0.15 mg
Duration of effective analgesia (min, mean ± SD)	136 ± 22	212 ± 72 ⱽ	193 ± 77 ⱽ	585 ± 446 ‡	333.6 ± 40.2	332.4 ± 36.6	684 ± 65.4 ‡
Duration of motor block (hours, median)	NR	NR	NR	NR	4 *	4 *	12
Duration of sensory block (min, mean ± SD)	NR	NR	NR	NR	NR	NR	NR

NR: not reported. * statistically significant: favours nalbuphine. ‡ statistically significant favours morphine. ⱽ statistically significant favours nalbuphine groups 0.8 mg and 1.6 mg compared to 0.2 mg.

**Table 5:** Secondary outcomes of studies comparing nalbuphine and morphine.

SECONDARY OUTCOMES	Culebras et al. (2000) [28]				Hassan et al. (2021) [31]		
	NALBUPHINE			MORPHINE	NALBUPHINE		MORPHINE
	0.2 mg	0.8 mg	1.6 mg	0.2 mg	1 mg	2 mg	0.15 mg
Onset of motor block (min, mean ± SD)	NR	NR	NR	NR	NR	NR	NR
Onset of sensory block (min, mean ± SD)	NR	NR	NR	NR	NR	NR	NR
Apgar score 1 min (median (IQR))	NS	NS	NS	NS	8.5 (7–9)	9 (8–10)	8 (7–9)
Apgar score 5 min (median (IQR))	NS	NS	NS	NS	9 (7–9)	9 (8–10)	9 (7–10)
Duration of complete analgesia (min, mean ± SD)	108 ± 23	176 ± 62 ⱽ	148 ± 45 ⱽ	275 ± 228 ‡	NR	NR	NR
**ADVERSE EFFECTS**							
Bradycardia (n,%)	NR	NR	NR	NR	NS	NS	NS
Hypotension (n,%)	NR	NR	NR	NR	NS	NS	NS
Shivering (n,%)	NR	NR	NR	NR	NR	NR	NR
Pruritus (n,%)	0 *	0 *	3 (13%) *	11 (50%)	2 (4%) *°	8 (16%) *	12 (24%)
PONV (n,%)	0	0	3 (13%)	5 (22.7%)	11 (22%)	20 (40%)	18 (36%)
Respiratory depression(n,%)	0	0	0	0	NR	NR	NR
Sedation (n,%)	NS	NS	NS	NS	NR	NR	NR

NR: not reported. NS: No actual values reported, but authors stated no statistically significant difference was detected. IQR: Interquartile range. * statistically significant: favours nalbuphine. ‡ statistically significant favours morphine. ⱽ statistically significant favours groups nalbuphine 0.8 mg and 1.6 mg compared to group nalbuphine 0.2 mg. ° statistically significant favours nalbuphine group 1 mg compared to nalbuphine 2 mg.

## Data Availability

The data supporting the findings of this study are available within the article and its Supplementary Materials.

## Supplementary Materials

The following supporting information can be downloaded at: https://www.mdpi.com/article/10.3390/jcm15114108/s1, PRISMA 2020 Checklist; File S1: GRADE assessment and Funnel plots for secondary outcomes.

## Abbreviations

The following abbreviations are used in this manuscript:

MD	Mean Difference
RR	Risk Ratio
PONV	Postoperative Nausea and Vomiting
PRISMA	Preferred Reporting Items for Systematic Reviews and Meta-Analyses
MeSH	Medical Subject Headings
LCSH	Library of Congress Subject Headings
LISTA	Library, Information, Science & Technology Abstracts
ASA	American Society of Anesthesiologists
RSS	Ramsay Sedation Scale
RevMan	Review Manager
CI	Confidence Interval
RoB 2	Revised Cohrane Risk of Bias Total for Randomised Trials
GRADE	Grading of Recommendations, Assessment, Development and Evaluation
RCT	Randomised Controlled Trial
VAS	Visual Analog Scale

## Appendix A

Search terms and Boolean operators used to identify relevant studies in electronic databases (Table A1).

**Table A1:** Search terms and Boolean operators.

DATABASE	SEARCH TERM SYNTAX
MEDLINE(PubMed)	((“Nalbuphine”[Mesh] OR nalbuphine[tiab]) AND (“Spinal Anaesthesia”[Mesh] OR “Regional Anaesthesia”[Mesh] OR intrathecal[tiab] OR subarachnoid[tiab] OR regional[tiab]))
Web of Science	TS = (nalbuphine) AND TS = (spinal OR intrathecal OR subarachnoid OR regional)
Scopus	(TITLE-ABS-KEY(nalbuphine) AND TITLE-ABS-KEY(spinal OR intrathecal OR subarachnoid OR regional))
Library of Congress	Subject: (Nalbuphine) AND (Spinal anaesthesia OR Intrathecal OR Subarachnoid OR Regional anaesthesia)
LISTA	(MH “Nalbuphine” OR TI nalbuphine OR AB nalbuphine) AND (MH “Spinal Anaesthesia” OR TI (intrathecal OR subarachnoid OR regional) OR AB (intrathecal OR subarachnoid OR regional))
