# Supplementary material for: Intrathecal Nalbuphine Versus Other Opioids as Adjuvant to 0.5% Hyperbaric Bupivacaine in Caesarean Section: A Systematic Review and Meta-Analysis
Source: J Clin Med. 2026 May 26;15(11):4108. doi: 10.3390/jcm15114108 (PMC13258391; doi:10.3390/jcm15114108)
Supplement: Supplementary file 1 [file jcm-15-04108-s001.zip › jcm-4209348-supplementary.pdf]

1. GRADE assessment

Certainty assessment							№ of patients		Effect		Certainty	Importance
№ of studies	Study design	Risk of bias	Inconsistency	Indirectness	Imprecision	Other considerations	intrathecal nalbuphine	intrathecal fentanyl	Relative (95% CI)	Absolute (95% CI)		
Duration of effective analgesia												
7	randomised trials	serious[a]	serious[b]	not serious	serious[c]	none	280	280	-	MD 13.1 minutes more (2.29 more to 23.9 more)	⊕◯◯◯ Very low[a,b,c]	CRITICAL
Duration of motor block												
8	randomised trials	very serious[d]	serious[e]	not serious	not serious	publication bias strongly suspected[f]	355	355	-	MD 6.41 minutes more (1.17 more to 11.65 more)	⊕◯◯◯ Very low[d,e,f]	CRITICAL
Duration of sensory block												
7	randomised trials	serious[a]	serious[e]	not serious	not serious	none	290	290	-	MD 1.95 minutes more (2.07 fewer to 6.83 more)	⊕⊕◯◯ Low[a,e]	CRITICAL
Onset of motor block												
7	randomised trials	serious[g]	serious[e]	not serious	not serious	none	280	280	-	MD 0.47 minutes more (0.16 more to 0.79 more)	⊕⊕◯◯ Low[e,g]	IMPORTANT

Onset of sensory block												
7	randomised trials	serious[a]	serious[e]	not serious	not serious	none	280	280	-	MD 0.44 minutes more (0.22 more to 0.67 more)	⊕⊕◯◯ Low[a,e]	IMPORTANT
Duration of complete analgesia												
3	randomised trials	serious[h]	serious[i]	not serious	serious[j]	none	110	110	-	MD 17.37 minutes more (2.38 more to 32.35 more)	⊕◯◯◯ Very low[h,i,j]	NOT IMPORTANT
Hypotension												
5	randomised trials	not serious	not serious	not serious	serious[k]	none	50/190 (26.3%)	55/190 (28.9%)	RR 0.92 (0.70 to 1.22)	23 fewer per 1.000 (from 87 fewer to 64 more)	⊕⊕⊕◯ Moderate[k]	IMPORTANT
Bradycardia												
6	randomised trials	serious[l]	not serious	not serious	serious[k]	none	7/230 (3.0%)	17/230 (7.4%)	RR 0.46 (0.17 to 1.22)	40 fewer per 1.000 (from 61 fewer to 16 more)	⊕⊕◯◯ Low[k,l]	IMPORTANT
PONV												
6	randomised trials	serious[l]	not serious	not serious	not serious	none	22/230 (9.6%)	44/230 (19.1%)	RR 0.53 (0.31 to 0.91)	90 fewer per 1.000 (from 132 fewer to 17 fewer)	⊕⊕⊕◯ Moderate[l]	IMPORTANT

Sedation												
3	randomised trials	not serious	not serious	not serious	not serious	none	120	120	-	MD 0.24 RSS lower (0.4 lower to 0.08 lower)	⊕⊕⊕⊕ High	IMPORTANT
Shivering												
5	randomised trials	serious[m]	not serious	not serious	not serious	none	10/180 (5.6%)	31/180 (17.2%)	RR 0.32 (0.16 to 0.64)	117 fewer per 1.000 (from 145 fewer to 62 fewer)	⊕⊕⊕◯ Moderate[m]	IMPORTANT
Pruritus												
6	randomised trials	serious[l]	serious[n]	not serious	serious[o]	none	2/230 (0.9%)	24/230 (10.4%)	RR 0.25 (0.08 to 0.86)	78 fewer per 1.000 (from 96 fewer to 15 fewer)	⊕◯◯◯ Very low[l,n,o]	IMPORTANT
Apgar Score at 1 min												
4	randomised trials	not serious	not serious	not serious	not serious	none	165	165	-	MD 0.01 units lower (0.17 lower to 0.15 higher)	⊕⊕⊕⊕ High	IMPORTANT

CI: confidence interval; MD: mean difference; RR: risk ratio

Explanations

a. Presence of one high risk of bias study and some concerns in 2 trials; however, sensitivity analysis excluding the high risk study do not materially change the results b. Substantial heterogeneity ($I^2 = 96\%$), although the direction of effect is consistent across most studies

c. The confidence interval is wide and includes both clinically trivial and important effects

d. High risk of bias in 2 studies, and sensitivity analysis shows loss of effect after exclusion of high risk trials

e. Serious heterogeneity ($I^2 > 90\%$), however the direction of effect is generally consistent with no clear differences in magnitudef. Assymetry in the funel plot

g. Presence of one high risk study and some concerns in two studies, with slight changes in the effect estimate after sensitivity analysis
h. One of the included studies is judged to be at high risk of bias and another with some concerns.
i. Substantial heterogeneity (I^2 =71%) and variability in effect estimates.
j. Confidence interval is wide and the total sample size is small, leading to uncertainty in the magnitude of effect
k. Confidence interval is wide and includes both potential benefit and harm, crossing the line of no effect.
l. One study is judged to be at high risk and two studies raise some concerns.
m. One study at high risk of bias and additional concerns in another study, which may affect the reliability of the pooled estimate.
n. Although overall statistical heterogeneity is low (I^2=8%), important differences between subgroups are observed, with inconsistent direction of effect across doses, suggesting potential clinical heterogeneity.
o. Confidence interval is wide and includes a range of clinically important effects, and the small number of events further contributes to uncertainty in the estimate.

2. Funnel plots for secondary outcomes

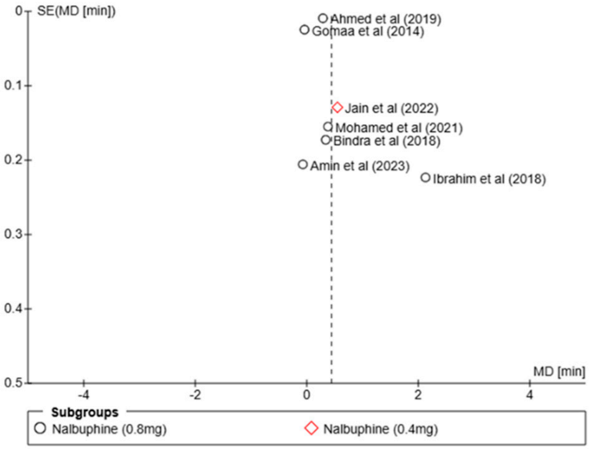

Funnel plot of studies examining onset time of sensory block in Nalbuphine (0.4mg or 0.8mg) versus Fentanyl (20mcg or 25mcg)

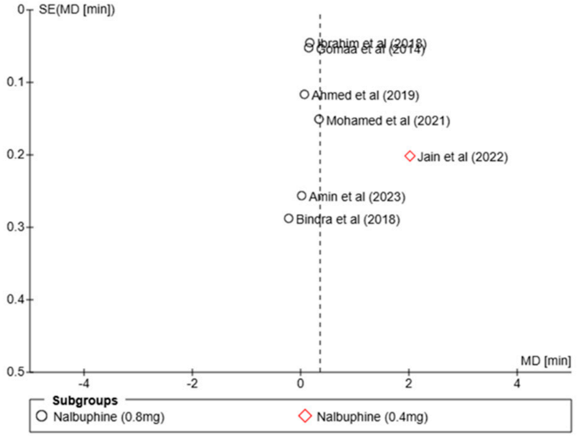

Funnel plot of studies examining onset time of sensory block in Nalbuphine (0.4mg or 0.8mg) versus Fentanyl (20mcg or 25mcg)

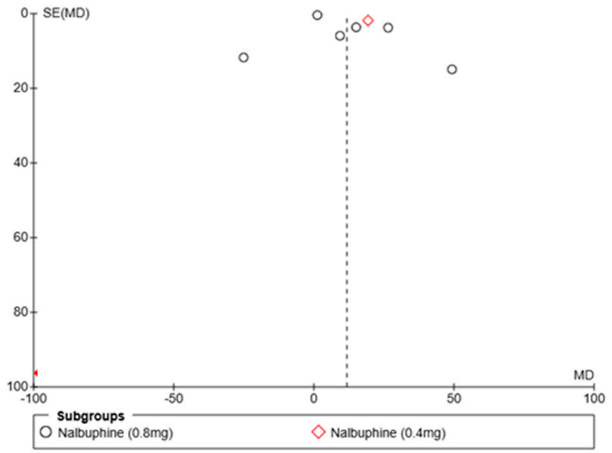

Funnel plot of studies examining bradycardia in Nalbuphine (0.4mg or 0.8mg) versus Fentanyl (20mcg or 25mcg)

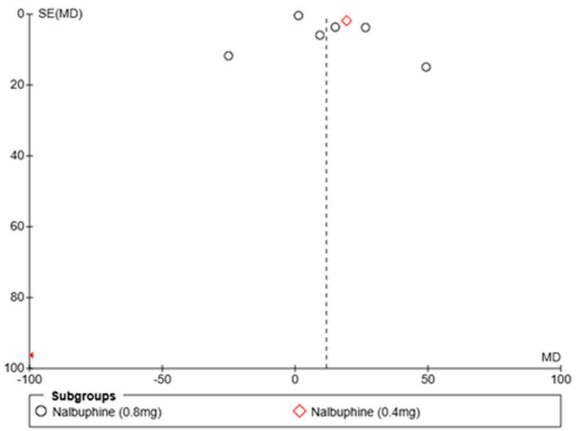

Funnel plot of studies examining hypotension in Nalbuphine (0.4mg or 0.8mg) versus Fentanyl (20mcg or 25mcg)

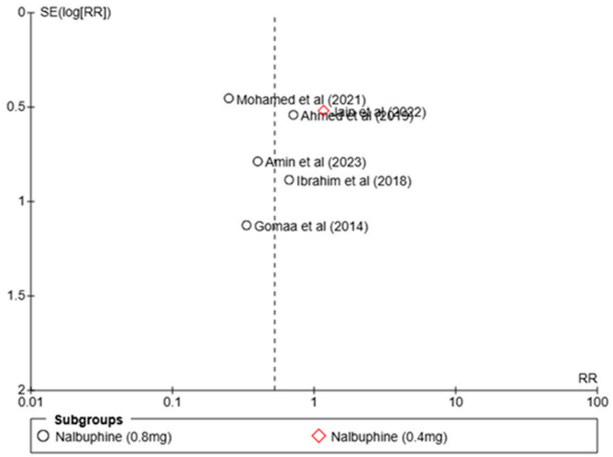

Funnel plot of studies examining PONV in Nalbuphine (0.4mg or 0.8mg) versus Fentanyl (20mcg or 25mcg)

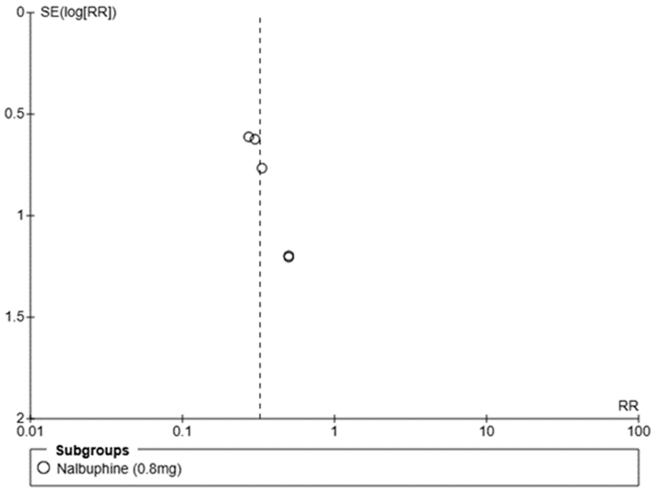

Funnel plot of studies examining shivering in Nalbuphine (0.8mg) versus Fentanyl (20mcg or 25mcg)

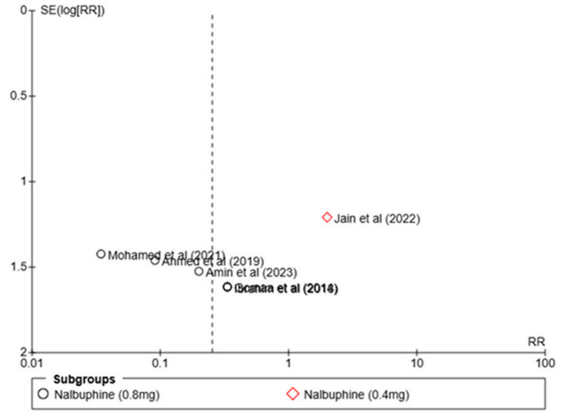

Funnel plot of studies examining pruritus in Nalbuphine (0.4mg or 0.8mg) versus Fentanyl (20mcg or 25mcg)

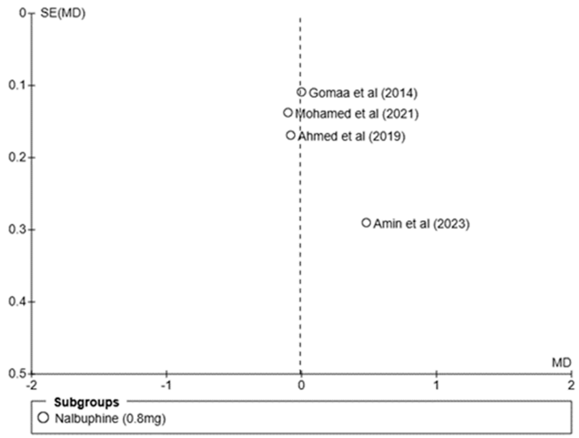

Funnel plot of studies examining APGAR score at 1 min in Nalbuphine (0.8mg) versus Fentanyl (20mcg or 25mcg)

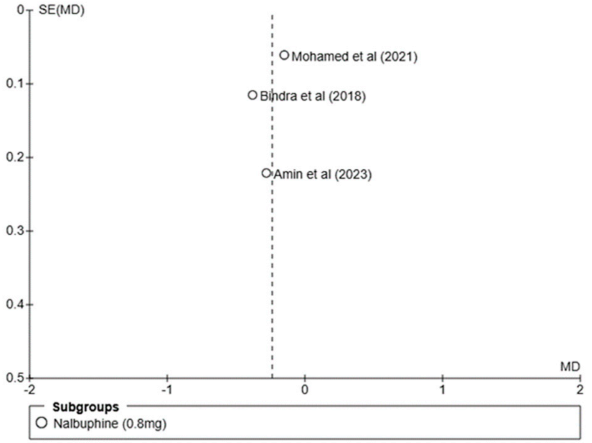

Funnel plot of studies examining sedation (RSS) in Nalbuphine (0.8mg) versus Fentanyl (20mcg or 25mcg)
